# Structural Determinants of Health Literacy Among Formerly Incarcerated Individuals: Insights From the Survey of Racism and Public Health

**DOI:** 10.3928/24748307-20241219-01

**Published:** 2025-01

**Authors:** Jemar R. Bather, Melody S. Goodman, Kimberly A. Kaphingst

## Abstract

**Background:**

Formerly incarcerated individuals (FIIs) encounter difficulties with covering the cost of dental and medical care, adhering to medication regimens, and receiving fair treatment from health care providers. Yet, no published research has examined modifiable pathways to increase FIIs' health literacy (HL), which is essential for addressing the health needs of this vulnerable population.

**Objective:**

The aim of this article is to examine neighborhood characteristics (neighborhood deprivation, racial and economic polarization, and residential segregation) and public assistance program enrollment as structural determinants of limited health literacy (LHL) among FIIs.

**Methods:**

Using a socioecological framework, we analyzed a subsample of 578 FIIs from the 2023 Survey of Racism and Public Health, an online cross-sectional survey spanning U.S. Department of Health & Human Services Regions 1, 2, and 3. HL was assessed using the Brief Health Literacy Screen. Logistic regression models estimated unadjusted and adjusted associations of LHL with neighborhood characteristics and public assistance program enrollment. Adjusted models controlled for age, race and ethnicity, gender identity, educational attainment, marital and employment status, number of children, chronic health conditions, and incarceration length.

**Key Results:**

The 578 FIIs had an average age of 46, with 42% having LHL. We observed a statistically significant association between public assistance program enrollment and LHL (unadjusted odds ratio [OR] = 2.72, 95% confidence interval [CI]: 1.87, 4.01; adjusted *OR* = 2.50, 95% CI: 1.62, 3.88). We found no statistically significant associations of LHL with neighborhood deprivation, racial and economic polarization, and residential segregation.

**Conclusions:**

Our findings suggest that there may be an opportunity to develop tailored interventions for increasing HL among FIIs through public assistance programs. Dissemination of HL resources among this marginalized group can improve their self-management of chronic diseases. This is of paramount importance because FIIs must simultaneously navigate other challenges after incarceration (e.g., unstable housing). [***HLRP: Health Literacy Research and Practice*. 2025;9(1):e8–e18.**]

## Abstract (plain language summary)

**Plain Language Summary: **We explored the HL of FIIs. We found that 42% had limited HL. FIIs enrolled in a public assistance program were more likely than those not enrolled to have limited HL, controlling for sociodemographic characteristics. We need to understand why these differences exist and work on ways to help increase health literacy among FIIs.

Federal and state legislation, like the First Step Act, has released nearly 2,000 individuals from prison daily (Carson, 2020; James, 2019). Extensive research shows that formerly incarcerated individuals (FIIs) simultaneously navigate several challenges during their return to society (Pager, 2003; Western, 2002; Western et al., 2015; Western & Beckett, 1999; Western & Wildeman, 2009). These challenges include the inability to secure adequate employment, obtain higher education credentials, and gain sufficient access to affordable and nutritious foods (Lindsay, 2022; Porter, 2019; A. Testa & Jackson, 2019; Wakefield & Uggen, 2010). FIIs also experience unstable housing, economic hardship, and marriage dissolution (Geller, 2013; Ginapp et al., 2023; Harris et al., 2010; Lopoo & Western, 2005; Martin et al., 2018; Massoglia et al., 2011; Turney, 2015). Consequently, FIIs prioritize addressing many of these challenges over effectively managing their health (Freudenberg et al., 2008). Research has demonstrated that FIIs exhibit a high prevalence of chronic diseases (e.g., asthma, hypertension), mental health conditions, hospitalization, and all-cause mortality (Houle, 2014; Howell et al., 2016; Massoglia, 2008; Massoglia et al., 2014; Rosen et al., 2011; Spaulding et al., 2011; Turney et al., 2012; Visher et al., 2004; Wang et al., 2009, 2013). FIIs encounter difficulties with covering the cost of dental and medical care, adhering to medication regimens, and receiving fair treatment from health care providers (Begun et al., 2016; Frank et al., 2014; Kulkarni et al., 2010). Yet, no published research has examined modifiable pathways to increase FIIs' health literacy, which is essential for addressing the health needs of this vulnerable population. Novel empirically based knowledge is needed to guide universal precautions, such as health literacy resources and strategies empowering FIIs to proactively self-manage their health.

Health literacy can be defined as “the degree to which individuals have the ability to find, understand, and use information and services to inform health-related decisions and actions for themselves and others” (Office of Disease Prevention and Health Promotion, n.d.). Limited health literacy has become a global public health priority due to its association with adverse health outcomes (Baker et al., 2007; Dewalt et al., 2004; Fan et al., 2016; Griffey et al., 2014; Paasche-Orlow et al., 2005; Qi et al., 2021; Schillinger et al., 2002). However, there has been a dearth of information concerning health literacy among FIIs. An analysis of FIIs in the Transitions Clinic Network showed that 60% of the study population had limited health literacy (Hadden et al., 2018). The authors of this study also reported that limited health literacy correlated with a higher probability of seeking emergency medical services and a lower confidence in taking prescribed medications (Hadden et al., 2018). Enhancing health literacy among FIIs is vital because it will help them better self-manage health, improve their quality of life, better interpret health communications, and increase medical decision-making knowledge (Berkman et al., 2010; Chen et al., 2014; Peerson & Saunders, 2009; Smith et al., 2015; Wetta et al., 2019).

Further investigations on health literacy among FIIs are needed to identify potential intervention points and inform innovative strategies and practical recommendations for increasing health literacy among this vulnerable population. Therefore, we draw on Schillinger's conceptual map depicting the interrelationships between social forces, health literacy, and health differences (Schillinger, 2020, 2021). Specifically, we focus on the pathway from social determinants of health to structural resources to health literacy (**Figure 1**). We conceptualize FIIs as a vulnerable subpopulation operating within structural mechanisms, which can be modified to enhance health literacy among this marginalized group.

**Figure 1.**
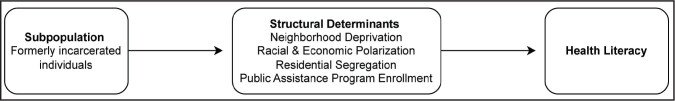
Conceptual model of neighborhood factors, public assistance program enrollment, and health literacy, Survey of Racism and Public Health, 2023.

We considered four structural components: public assistance program enrollment, neighborhood deprivation, racial and economic polarization, and residential segregation. Prior work in this area has focused on the efficacy of enrolling individuals in Medicaid before their release and states implementing the Medicaid expansion policy (Blackburn et al., 2020; Burns et al., 2021, 2022; Wennerstrom et al., 2023), which deemed FIIs eligible for receiving health care coverage (Howard et al., 2016). Analyzing the relationship between public assistance program enrollment and health literacy is crucial because it provides an intervenable pathway to incorporate engaging and sustainable strategies for increasing FIIs' health literacy. To our knowledge, neighborhood characteristics and health literacy among FIIs has received far less attention. The deleterious relationship between disadvantaged neighborhoods and health outcomes is well-documented (Bailey et al., 2017; Braveman et al., 2022; M. Goodman et al., 2018; Kramer & Hogue, 2009; Phelan & Link, 2015; White & Borrell, 2011; Williams et al., 2019; Yearby, 2020). Identifying neighborhoods as a determinant of health literacy among FIIs is relevant to health communication scientists who can devise cluster randomized trials at the neighborhood level to assess the impact of health literacy campaigns among individuals with a criminal record.

Building on prior research (Blackburn et al., 2020; Burns et al., 2021, 2022; Hadden et al., 2018; Howard et al., 2016; Wennerstrom et al., 2023), the present study's objective was to examine neighborhood characteristics (neighborhood deprivation, racial and economic polarization, and residential segregation) and public assistance program enrollment as determinants of limited health literacy among FIIs (**Figure 1**). We explored associations of these modifiable pathways using a subsample of over 500 FIIs from the 2023 Survey of Racism and Public Health (Bather et al., 2024). We hypothesized that FIIs living in areas with greater social disadvantage would have increased odds of having limited health literacy. This empirical analysis will be informative for public health scientists, health communication specialists, legal epidemiologists, and policymakers working to mitigate the highly prevalent chronic diseases among this vulnerable population (Binswanger et al., 2009; Kulkarni et al., 2010; Leukefeld et al., 2006; Mallik-Kane & Visher, 2008; Pizzicato et al., 2018; Ranapurwala et al., 2018).

## Methods

### Participant Recruitment and Data Collection

A multidisciplinary team of statisticians, applied psychologists, epidemiologists, and community health scientists at the Center for Anti-racism, Social Justice & Public Health (CASJPH) hired Qualtrics Research Services (QRS) to field the Survey of Racism and Public Health (SRPH). QRS is an online research panel service that compensates (e.g., gift cards) its panel members for survey participation. QRS recruits panel members through various mechanisms, such as targeted marketing materials, referrals, and conferences. The multidisciplinary scientists at the CASJPH provided QRS with institutional access to a Qualtrics account to recruit study participants, manage survey data collection, and provide incentives to study participants. QRS is a third-party service that had no relationship with the CASJPH researchers. The CASJPH scientists also did not interact with or recruit any study participants. The SRPH is an online cross-sectional survey that took participants about 15 minutes to complete. The SRPH aimed to learn more about participants' experiences with discrimination, interactions with the police, financial and food security, voting practices, and health. Readability was assumed for all survey measures. The study participants were not provided with assistance to complete any of the survey components. The primary analyses intended for the SRPH did not focus on FIIs as a vulnerable subpopulation. We chose the SRPH for this secondary data analysis because of the availability of incarceration history, zip code information, and health literacy data.

QRS recruited potential participants who were at least age 18 years, English proficient, and resided in states/territories within U.S. Department of Health & Human Services Regions 1, 2, and 3. These regions encompassed Connecticut, Delaware, the District of Columbia, Maine, Maryland, Massachusetts, New Hampshire, New Jersey, New York, Pennsylvania, Puerto Rico, Rhode Island, Vermont, and Virginia (Region 1). The U.S. Virgin Islands (Region 2) and West Virginia (Region 3) also comprise regions of the U.S. Department of Health & Human Services but were not included as recruitment areas because these areas were not within scope of the primary analyses for the SRPH. QRS oversampled minoritized racial and ethnic groups (50% White, 20% Black, 20% Latino/a/e, and 10% American Indian/Native American, Arab/Middle Eastern/North African, Asian American/Pacific Islander, and Multiracial) and obtained specific age group distributions (30% aged 18–34, 32% aged 35–54, and 38% aged 55 or older). Study recruitment began in March 2023 and ended in April 2023 once target distributions were achieved for race and ethnicity and age. Additional details on the study protocol have been reported previously (Bather et al., 2024). Informed consent was obtained prior to survey participation. The New York University Institutional Review Board approved the study protocol (IRBFY2023-7408). The present analysis adheres to the Declaration of Helsinki and the Strengthening the Reporting of Observational Studies in Epidemiology guidelines (von Elm et al., 2007).

### Analytic Sample

**Figure 2.**
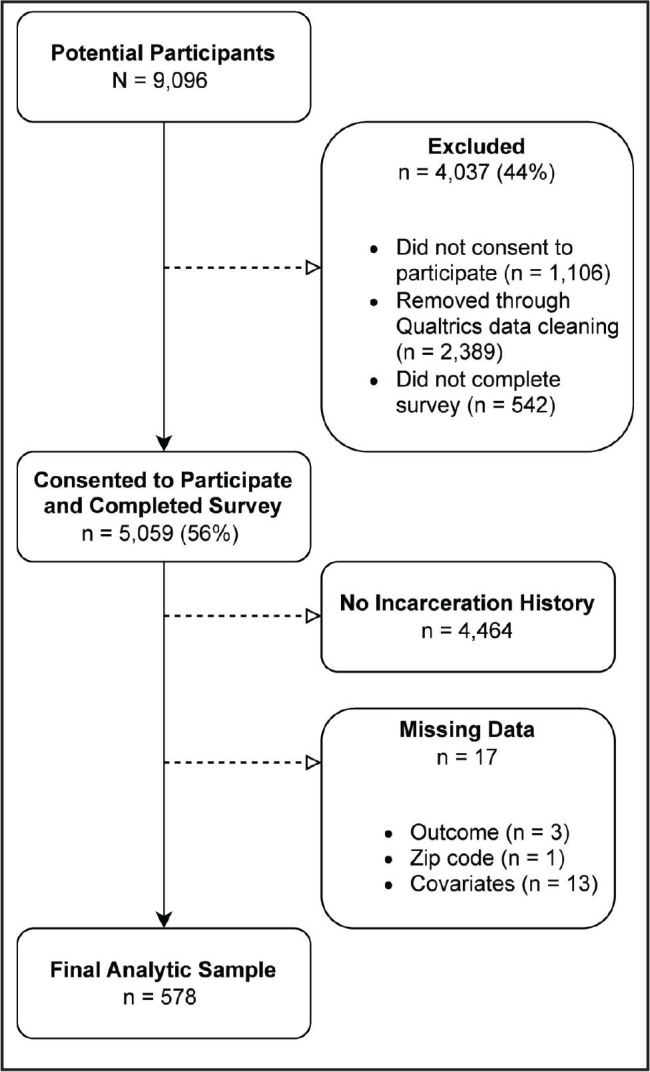
Analytic sample flow diagram, Survey of Racism and Public Health, 2023.

Between March and April 2023, 9,096 potential participants were invited to complete the SRPH. Among the initial 9,096 potential participants (**Figure 2**), 1,106 (12%) declined to participate, 542 (6%) did not finish the survey, and 2,389 (26%) were excluded through Qualtrics data cleaning services. Reasons for exclusion included invalid IP addresses, nonsensical responses, implausible height or weight values, duplications, contradictory answers, and bot activity. This resulted in 5,059 participants who both consented and completed the survey. Participants were further excluded based on their binary responses (yes or no) to the question: “Have you ever been incarcerated?” This process identified 595 participants who self-reported having an incarceration history. We then excluded 17 (3%) participants due to missing information regarding health literacy, zip code, or covariates, yielding a final analytic sample of 578 FIIs.

### Health Literacy Assessment

Health literacy was assessed using the Brief Health Literacy Screen (BHLS), a validated subjective measure that has been applied among population-based samples, primary care patients, and a nationwide cohort of young women with breast cancer (Kaphingst et al., 2014, 2021; Odumegwu et al., 2024; Waters et al., 2018). The BHLS comprises three Single Item Literacy Screeners assessing a participant's difficulty reading hospital materials, difficulty learning about their medical condition, and confidence in filling out medical forms (Carpenter et al., 2014; Chew et al., 2008; M. S. Goodman et al., 2015). Response choices for the difficulty questions ranged from (0) *none of the time* to (4) *all of the time*, and the confidence question had response options from (0) *not at all* to (4) *extremely*. To create a composite BHLS index (range: 0–11), responses to the confidence question were reverse-coded and summed with the responses to the difficulty questions. Index scores of three or higher were coded as having limited health literacy, and scores lower than three were coded as having adequate health literacy. This dichotomization is consistent with previous health literacy studies (Carpenter et al., 2014; Fan et al., 2016). We computed the alpha reliability coefficient (Cronbach's *α* = 0.64) for the BHLS.

### Neighborhood Factors

We merged self-reported zip codes with census tract-level measures of neighborhood deprivation, racial and economic polarization, and residential segregation. We created weighted measures by multiplying census tract weights within each zip code to each neighborhood characteristic. We obtained neighborhood measures using the ndi R package (Buller, 2022) and census tract weights using US Department of Housing and Urban Development US Postal Service Zip Code CrossWalk files (HUD Office of Policy Development and Research, n.d.). We standardized each weighted neighborhood measure to have a mean of 0 and a standard deviation of 1.

We assessed neighborhood deprivation using the Powell-Wiley Neighborhood Deprivation Index (NDI) (Andrews et al., 2020; Slotman et al., 2022), which is based on a factor analysis of 13 socioeconomic factors (e.g., education, occupation) (Diez Roux & Mair, 2010). Theoretical NDI scores range from −3.6 to 2.8, with higher scores indicating greater neighborhood deprivation (Andrews et al., 2020; Slotman et al., 2022). We used the Index of Concentration at the Extremes (ICE) to measure racial and economic polarization (Feldman et al., 2015; Krieger et al., 2016). ICE scores are derived from racial and ethnic stratified household income estimates, theoretically ranging from −1 to 1. A score of −1 denotes a complete concentration of residents racialized as Black with low income (Feldman et al., 2015; Krieger et al., 2016). By contrast, a score of 1 represents a complete concentration of residents racialized as White with high income (Feldman et al., 2015; Krieger et al., 2016). We assessed residential segregation using the Dissimilarity Index (DI) (Duncan & Duncan, 1955). Theoretical DI scores range from 0 to 1, representing the proportion of individuals racialized as Black needed to relocate to achieve an equal Black-to-White distribution (Duncan & Duncan, 1955).

### Public Assistance Program Enrollment

The survey collected information on enrollment in public assistance programs. Participants were asked: “Have you received public assistance in the last 12 months? Please select all that apply.” Response options included: Disability; Medicaid; Supplemental Nutrition Assistance Program; Temporary Assistance for Needy Families; Unemployment; Special Supplemental Nutrition Program for Women, Infants, and Children; other; and none. Responses were summed for each participant and categorized as zero versus one or more programs.

### Sociodemographic Characteristics

We controlled for several key sociodemographic characteristics based on prior literature (Al Sayah et al., 2013; Berkman et al., 2011; Easton et al., 2010; Paasche-Orlow & Wolf, 2007). These following characteristics were recategorized for analysis: age (measured continuously), race and ethnicity (White, Black, Latino/a/e, American Indian/Native American, Arab/Middle Eastern/North African, Asian American/Pacific Islander, and Multiracial), gender identity (man, woman), educational attainment (high school or less, some college, college degree or higher), marital status (married/living with a partner, divorced/separated/widowed, never married), employment status (full-time, part-time, independent contractor/business owner, looking for work/unemployed), number of children (0, 1, 2+), number chronic health conditions (0, 1+), and incarceration length (less than 1 year, 1 year or more). One item was used to assess participants' number of chronic health conditions: “Have you ever been diagnosed with any of the following conditions? Check all that apply.” Response options included: arthritis, asthma, cancer, chronic lung disease, congestive heart failure, diabetes mellitus, periodontal disease, hypertension, mental health condition/psychiatric problems, obesity, stroke, substance use disorder, and none. Responses were summed and categorized as zero versus one or more chronic health conditions.

### Statistical Analysis

Data were analyzed using R (R Core Team, 2023), with statistical significance set at *p* < .05. We used the gtsummary R package to examine descriptive statistics for all variables (Sjoberg et al., 2021). Bivariate relationships with health literacy were evaluated using the Wilcoxon rank sum and Pearson's Chi-squared tests. Logistic regression models estimated unadjusted and adjusted associations with health literacy. We present odds ratios with 95% confidence intervals.

## Results

### Description of Sample

The 578 FIIs had an average age of 46 (standard deviation [*SD*] = 14), with 42% having limited health literacy based on the BHLS (**Table 1**). The majority identified as White (44%) or Black (28%) and as a man (69%). Sixty-one percent had at least some college education, which includes those who earned some college credits, an associate degree, a bachelor's degree, or a graduate degree. Almost half were married or living with a partner (47%), working full-time (47%), and had no children (46%). Seventy percent were previously incarcerated for less than a year, 80% had at least one chronic health condition, and 68% were enrolled in at least one public assistance program. The average NDI score was 0.3 (*SD* = 0.8), indicating that participants tended to reside in areas with moderate neighborhood deprivation. The mean ICE score was 0.1 (*SD* = 0.2), suggesting that, on average, study participants did not live in areas of high racial and economic polarization. The average DI score was 0.3 (*SD* = 0.1), indicating that participants tended to live in areas that require 30% of individuals racialized as Black to move to achieve an equal Black-to-White distribution. **Table 1** also shows statistically significant differences in characteristics by health literacy status. Compared to those with adequate health literacy (AHL), FIIs with limited health literacy (LHL) were more likely to be enrolled in at least one public assistance program (LHL: 80%; AHL: 59%; *p* < .001), have two or more children (LHL: 39%; AHL: 23%; *p* < .001), and have an incarceration history of one year or more (LHL: 37%; AHL: 24%; *p* = .001).

**Table 1:**
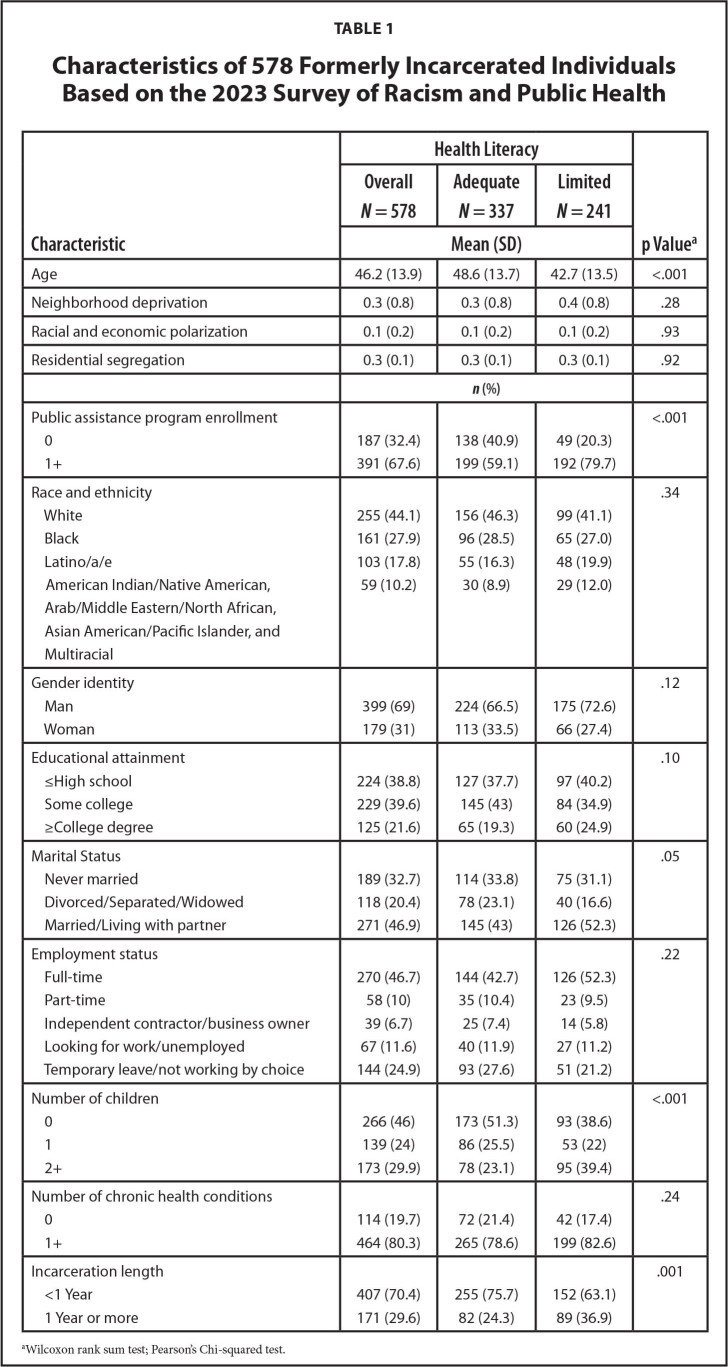
Characteristics of 578 Formerly Incarcerated Individuals Based on the 2023 Survey of Racism and Public Health

**Characteristic**	**Health Literacy**			**p Value^a^**

	**Overall *N* = 578**	**Adequate *N* = 337**	**Limited *N* = 241**	

	**Mean (SD)**			

Age	46.2 (13.9)	48.6 (13.7)	42.7 (13.5)	<.001

Neighborhood deprivation	0.3 (0.8)	0.3 (0.8)	0.4 (0.8)	.28

Racial and economic polarization	0.1 (0.2)	0.1 (0.2)	0.1 (0.2)	.93

Residential segregation	0.3 (0.1)	0.3 (0.1)	0.3 (0.1)	.92

	***n* (%)**			

Public assistance program enrollment				<.001
0	187 (32.4)	138 (40.9)	49 (20.3)	
1+	391 (67.6)	199 (59.1)	192 (79.7)	

Race and ethnicity				.34
White	255 (44.1)	156 (46.3)	99 (41.1)	
Black	161 (27.9)	96 (28.5)	65 (27.0)	
Latino/a/e	103 (17.8)	55 (16.3)	48 (19.9)	
American Indian/Native American,	59 (10.2)	30 (8.9)	29 (12.0)	
Arab/Middle Eastern/North African,				
Asian American/Pacific Islander, and Multiracial				

Gender identity				.12
Man	399 (69)	224 (66.5)	175 (72.6)	
Woman	179 (31)	113 (33.5)	66 (27.4)	

Educational attainment				.10
≤High school	224 (38.8)	127 (37.7)	97 (40.2)	
Some college	229 (39.6)	145 (43)	84 (34.9)	
≥College degree	125 (21.6)	65 (19.3)	60 (24.9)	

Marital Status				.05
Never married	189 (32.7)	114 (33.8)	75 (31.1)	
Divorced/Separated/Widowed	118 (20.4)	78 (23.1)	40 (16.6)	
Married/Living with partner	271 (46.9)	145 (43)	126 (52.3)	

Employment status				.22
Full-time	270 (46.7)	144 (42.7)	126 (52.3)	
Part-time	58 (10)	35 (10.4)	23 (9.5)	
Independent contractor/business owner	39 (6.7)	25 (7.4)	14 (5.8)	
Looking for work/unemployed	67 (11.6)	40 (11.9)	27 (11.2)	
Temporary leave/not working by choice	144 (24.9)	93 (27.6)	51 (21.2)	

Number of children				<.001
0	266 (46)	173 (51.3)	93 (38.6)	
1	139 (24)	86 (25.5)	53 (22)	
2+	173 (29.9)	78 (23.1)	95 (39.4)	

Number of chronic health conditions				.24
0	114 (19.7)	72 (21.4)	42 (17.4)	
1+	464 (80.3)	265 (78.6)	199 (82.6)	

Incarceration length				.001
<1 Year	407 (70.4)	255 (75.7)	152 (63.1)	
1 Year or more	171 (29.6)	82 (24.3)	89 (36.9)	

a Wilcoxon rank sum test; Pearson's Chi-squared test.

### Associations with Limited Health Literacy

**Table 2** shows the unadjusted and adjusted relationships with limited health literacy among FIIs in this sample. We observed a statistically significant association between public assistance program enrollment and limited health literacy (unadjusted *OR* = 2.72, 95% CI: 1.87, 4.01; adjusted *OR* = 2.50, 95% CI: 1.62, 3.88). The adjusted model controlled for age, race/ethnicity, gender identity, educational attainment, marital status, employment status, number of children, chronic health conditions, and incarceration length. We found no statistically significant associations of limited health literacy with neighborhood deprivation, racial and economic polarization, and residential segregation.

**Table 2:**
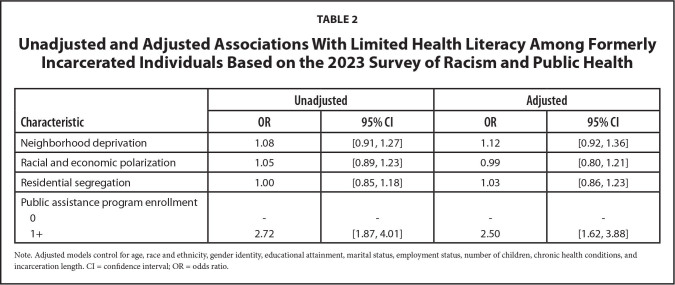
Unadjusted and Adjusted Associations With Limited Health Literacy Among Formerly Incarcerated Individuals Based on the 2023 Survey of Racism and Public Health

**Characteristic**	**Unadjusted**		**Adjusted**	

	**OR**	**95% CI**	**OR**	**95% CI**

Neighborhood deprivation	1.08	[0.91, 1.27]	1.12	[0.92, 1.36]

Racial and economic polarization	1.05	[0.89, 1.23]	0.99	[0.80, 1.21]

Residential segregation	1.00	[0.85, 1.18]	1.03	[0.86, 1.23]

Public assistance program enrollment				
0	-	-	-	-
1+	2.72	[1.87, 4.01]	2.50	[1.62, 3.88]

Note. Adjusted models control for age, race and ethnicity, gender identity, educational attainment, marital status, employment status, number of children, chronic health conditions, and incarceration length. CI = confidence interval; OR = odds ratio.

## Discussion

This study explored the associations of neighborhood factors and public assistance program enrollment with limited health literacy among FIIs. The data showed that FIIs enrolled in at least one public assistance program were more likely than those not enrolled to have limited health literacy, controlling for sociodemographic characteristics. We did not observe any statistically significant associations of limited health literacy with neighborhood deprivation, racial and economic polarization, and residential segregation.

To our knowledge, this is the first study to examine the relationships between neighborhood factors, public assistance program enrollment, and limited health literacy among FIIs. Hadden et al. (2018) studied 751 FIIs in the Transitions Clinic Network, encompassing six states and Puerto Rico. They found that FIIs with limited health literacy tended to visit the emergency department more frequently and have lower confidence in taking medications after release than FIIs with adequate health literacy (Hadden et al., 2018). The findings from the present study add to prior literature, showing that FIIs enrolled in at least one public assistance program tended to have limited health literacy. Following Schillinger's socio-ecological framework (Schillinger, 2020, 2021), the observations from the present study suggest that public assistance programs may be an opportunity to develop targeted interventions for increasing health literacy among FIIs. Higher health literacy may decrease this vulnerable population's high chronic disease incidence (Schnittker et al., 2012; Schnittker & John, 2007; Udo, 2019; Wang et al., 2017; Wang & Green, 2010; Wilper et al., 2009). This is of paramount importance because FIIs must simultaneously navigate other challenges after incarceration (e.g., unstable housing) (Apel et al., 2010; Evans et al., 2019; Harper et al., 2021; Larson et al., 2022; Leasure & Martin, 2017; Maroto, 2015; Santos et al., 2023; Widdowson et al., 2020).

Program enrollment timing may be a plausible explanation for observing a significant association between public assistance program enrollment and limited health literacy. It may be that most of the study population categorized as enrolled in a public assistance program may have enrolled during post-release. Future investigations should evaluate whether associations of public assistance program enrollment with limited health literacy differ between those who enrolled before and after release. Collectively, findings from these studies can inform policies aimed at enrolling incarcerated individuals in public assistance programs before their release, which, in turn, may positively affect their health. An example is the Medicaid expansion, which grants eligibility to FIIs for health care coverage (Howard et al., 2016). In a cohort study of 16,000 FIIs with substance use history, Burns et al. (2022) observed increased utilization of outpatient care services after Wisconsin established a pre-release Medicaid enrollment assistance program. Relatedly, other researchers have detected significant increases in Medicaid enrollment following the implementation of a pre-release Medicaid enrollment assistance program (Blackburn et al., 2020; Burns et al., 2021; Wennerstrom et al., 2023).

The nonsignificant association between neighborhood characteristics and limited health literacy aligns with prior research, showing that past neighborhood characteristics have a greater impact on health literacy, genetic knowledge, and upward mobility than current neighborhood characteristics (Bather et al., 2023; Chetty et al., 2016, 2020; Chetty & Hendren, 2018). The present study assessed neighborhood factors using self-reported zip codes. Longitudinal analyses are needed to ascertain whether past neighborhood characteristics impact health literacy among FII more than current neighborhood characteristics.

## Limitations and Strengths

This study has several limitations. First, we observed a low reliability coefficient (0.64) for the BHLS, likely due to differing samples between this study and the BHLS validation sample (Chew et al., 2008). The initial analyses planned for the Survey of Racism and Public Health did not specifically target FIIs as a vulnerable subgroup. However, we selected the SRPH for this secondary analysis due to the availability of information on participants' incarceration history, residential zip codes, and BHLS measure. We used a self-report subjective measure of health literacy. Future analyses should assess health literacy among FIIs using a test-based health literacy measure. Second, the nonprobability cross-sectional survey design limits generalizability because all the states considered for recruitment are in the eastern region of the United States. Furthermore, the analytic sample was not representative of the formerly incarcerated population. Longitudinal studies using generalizable designs are needed to draw causal inferences and account for trends over time. Third, self-reporting incarceration history may have been sensitive for some participants, possibly leading to underreporting of incarceration history, thus excluding some FIIs from the analysis.

Our study has several strengths despite these limitations. We used Schillinger's socio-ecological framework to examine structural determinants of limited health literacy among FIIs (Schillinger, 2020, 2021). We used a subsample of over 500 FIIs from the Survey of Racism and Public Health, a unique data source spanning U.S. Department of Health & Human Services Regions 1, 2, and 3. We used validated measures of neighborhood deprivation, racial and economic polarization, residential segregation, and health literacy, which can be expanded and developed for use in international settings. The current investigation used census weights to account for different zip codes covered within a census tract.

## Conclusions

The overall prevalence of limited health literacy among FIIs is high. Still, the practical resources and strategies to reduce limited health literacy among this vulnerable population remain inconclusive. Using a socioecological framework, modifiable pathways (neighborhood factors and public assistance program enrollment) in relation to limited health literacy. We found that FIIs enrolled in at least one public assistance program were more likely to have limited health literacy than those not enrolled. Implications for public health and clinical practice include developing tailored interventions for increasing health literacy through public assistance programs and disseminating resources among FIIs to help with the self-management of chronic diseases.
